# Designing and conducting MD/MPH dual degree program in the Medical School of Shiraz University of Medical Sciences

**Published:** 2015-07

**Authors:** ALIREZA SALEHI, NEDA HASHEMI, MAHBOOBEH SABER, MOHAMMAD HADI IMANIEH

**Affiliations:** 1 MPH Department, Medical School, Shiraz University of Medical Sciences, Shiraz, Iran;; 2 Department of Health Management and Economics, Public Health School, Tehran University of Medical Sciences, Tehran, Iran;; 3 Medical Ethics Department, Medical School, Shiraz University of Medical Sciences, Shiraz, Iran;; 4 Research Center for Traditional Medicine and History of Medicine, Medical School, Shiraz University of Medical Sciences, Shiraz, Iran

**Keywords:** Medical education, Public health, Organization, Administration

## Abstract

**Introduction:**

Many studies have focused on the need of health systems to educated physicians in the clinical prevention, research methodology, epidemiology and health care management and emphasize the important role of this training in the public health promotion. On this basis, Shiraz University of Medical Sciences (SUMS) has established MD/MPH dual degree program since the year 2012.

**Methods:**

In the current study, Delphi technique was used. Both qualitative and quantitative methods were applied in the Delphi process. The Delphi team members including experts with extensive experience in teaching, research and administration in the field of educational management and health/medical education reached consensus in almost 86% of the questionnaire items through three Delphi rounds. MD/MPH program for SUMS was designed based on the items agreed and thematic analysis used in these rounds.

**Results:**

The goals, values, mission and program requirements including the period, the entrance condition, and the number of units, and certification were determined. Accordingly, the courses of the program are presented in parallel with the MD education period. MPH courses consist of 35 units including 16 obligatory and 15 voluntary ones.

**Conclusion:**

Designing MD/MPH program in SUMS based on the existent models in the universities in different countries, compatible with educational program of this university and needs of national health system in Iran, can be a beneficial measure towards promoting the students’ knowledge and theoretical/practical skills in both individual and social level. Performing some additional research to assess the MD/MPH program and some cohort studies to evaluate the effect of this program on the students’ future professional life is recommended.

## Introduction

According to the views of many experts, students' education in most of the medical schools is focusing on the individual treatment and diagnosis based on the biomedical model; thus, the absence or lack of education emphasizing the population-based model and prevention is considerable. Remarkably, the estimations show that less than 1% of physicians passing adequate training in the health sciences and having inadequate knowledge not only imposes extra costs on the health system, but also has a detrimental effect on the quality of research and clinical practice (1-3).

Accordingly, many studies indicate the need of health systems to well-educated physicians in the clinical prevention, research methodology, epidemiology and health care management and emphasize the important role of this training in the public health promotion (4-10). Most of the well-known universities in the world have merged master of public health (MPH) with medicine and only in the United States of America 24 accredited universities provide this integrated degree program (11). Yale, Stanford, Boston, Illinois and Harvard universities are some of them (12-16).

In Iran, Tehran University of Medical Sciences (TUMS) established the MPH dual degree program for the first time in the year 2007-8 by admitting 42 exceptionally talented students. The main goal of this program is training students in analytical and managerial skills needed for development of expert managers in clinical and public health fields and its emphasis is on the functional solution of health system issues. 

The most important primary motivations of students who entered this program in different countries were learning research methodology scientifically and systematically, improving academic resume for post-graduate education and achieving academic or managerial position (12). General evaluations and feedbacks of the program in different countries show that it has been successful in achieving the designers' and participants' objectives (1, 3, 17-20).

On this basis, Shiraz University of Medical Sciences (SUMS) established MD/MPH dual degree program in the year 2012. This article describes the design phases of this period as well as its details. 

## Methods

In the current study, Delphi technique was used. Both qualitative and quantitative methods were applied in the Delphi process. Delphi is a structured process that uses a series of questionnaires or ‘rounds’ to gather information until consensus in the panels is reached (21). This technique is useful for situations where individual judgments must be combined in order to address a lack of agreement or incomplete state of knowledge, as was the case for this research (22, 23). To form the Delphi team, 23 experts were identified. All of these professionals were university faculties and had extensive experience in teaching, research and administration in the field of educational management and health/medical education. 23 mails including explanations about research rounds and goals along with an invitation to participate in the study were sent to each individual. 19 of invitees stated their agreement to participate in the study. Therefore, they were selected as Delphi team members (Table 1). The Delphi rounds were as follows:

**Table 1:** Characteristics of Delphi team members

	**Classification**	**N (%)**
**Gender**	Male	15 (79)
	Female	4 (21)
**Academic position**	Instructor	2 (11)
	Assistant professor	3 (16)
	Associate professor	9 (47)
	Professor	5 (26)
**Job position**	Vice Chancellor	2 (10)
	Dean/ Deputy	6 (32)
	Head of the department / Assistant for educational affaire	7 (37)
	Research center director	4 (21)
**Age**	Mean±SD	53±4.3

***First round***

The first questionnaire consisted of 26 questions obtained from literature reviews and expert members’ opinions of the committee responsible for designing and implementing the program. To allow expression of a wide range of views, the questionnaire comprised open-ended questions (24). Printed copies of the questionnaire were distributed among Delphi team members and collated after a specific time period.

Simultaneously, the Latin databases including Medline, Cochrane, Google Scholar, Scirus, Embase, and Web of Science were searched and related studies, rules and guidelines on the topic of instructional system design, MD/MPH program and dimensions of educational programs applied in different universities throughout the world were gathered. After the primary search, a number of secondary searches were also conducted based upon “related links” as well as additional works by the authors identified in the primary search to gather excessive data. 

Thereafter, generated ideas and suggestions from round 1 and gathered data from literature review were combined and similar ideas were clustered into emerging themes. Three of the authors as the Delphi coordinators did this separately at first and then jointly to discuss different interpretations. The items were used as input for round 2. 

***Second round***

In the second round, the participants were presented with the previous round items and themes. The participants were asked to score each item using five-point Likert scale (extremely important=5, very important=4, moderately important=3, slightly important=2, not important=1). Space was provided for optional comments at the end of each theme and at the end of instrument. Based on the literature, we defined consensus as at least 80% of the participants in the Delphi team ticking the same answer category (e.g. 5 ‘extremely important’) and no more than 15% an answer category two or three categories away (e.g. 2 ‘slightly important’ or 1 ‘not important’) (21). Items on which consensus was reached were removed from the subsequent questionnaire(s).

***Third round***

In the third questionnaire, the other items on which the consensus was not achieved in the previous round were included, together with feedback on the responses of the panel and the participant’s own responses. The participants were asked to reconsider their previously given responses in light of the opinion of other panel members. Space was again made available at the end of each theme as well as at the end of the instrument for optional comments. The scoring process was the same as the previous round. At the end of the round, consensus was reached in almost 86% of the questionnaire items. Therefore, the Delphi team members reached consensus and Delphi rounds were stopped.

Afterwards, MD/MPH program for SUMS was designed based on the agreed items and thematic analysis used in the Delphi rounds. This program model was recommended to the competent authorities in the university and was assessed. Finally, the suggested program was approved by the university authorities and got started.

## Results

**Goals of the program**

The general goal of the MD/MPH program is promoting the students' analytical skills and preparing them to participate in a wide range of positions in the health and clinical care. The specific goals of the program are as follows:

- The development of the students' managerial skills to cope with future health managerial duties
- The development of the students' analytical skills to enable them to practically resolve the problems of the health system
- The development of the students' community-based skills
- The development of the student*s*' research skills
- The improvement of the student*s*' creativity and innovation

**Values of the program**

Values of the MD/MPH program at SUMS are as follows:

1. The maintenance and promotion of the communities' health, based on the human rights
2. Justice in health
3. Community based health
4. Enabling people to manage their health care
5. Accountability, responsibility and approaching health as a human right by health care providers

**The mission of the program**

The students should attain the following abilities after passing MD/MPH Program:

- Identifying the health issues at national and international level accurately
- Determining the scope and size of the health issues
- Determining the reasons and factors influencing health
- Designing appropriate strategies for prevention and intervention
- Determining the most effective method of intervention based on the political, economic and social situation
- Developing the new methods of health care management and its evaluation

**The period of the program**

Medical education in Iran lasts 7 years and consists of 3 stages including basic sciences, physiopathology, and clerkship. The students enter the MPH program at the beginning of the physiopathology stage. The courses of the program are presented in parallel with the MD education period. 

**Conditions for entrance to the program**

Top medical students whose grade point average of their previous semesters is A (17/20 or higher) are eligible to enter the MD/MPH program. After the students register in the program voluntarily, the written and oral exams will be held. The students meeting the passing score will be eligible to enter the MD/MPH program.

**The number of the courses**

MPH courses consist of 35 units. 16 obligatory units are offered during the first stage and 15 voluntary units are offered during the second stage of the program. The voluntary units are selected based on the students’ interests. Thesis as a 4 unit course should be passed at the end of the program. Its topic is chosen based on the health system/university research priorities and students’ interest (Table 2).

**Table 2:** The obligatory and voluntary MD/MPH courses

	**Courses**	**Units**
**obligatory**	The principals of epidemiology and research methodology	3
	Applied biostatistics	2
	Advanced epidemiology	2
	Statistical methods	3
	The epidemiology of communicable disease	2
	The epidemiology of non-communicable disease	2
	Nutritional epidemiology	2
	Thesis	4
	Total	20
**Voluntary**	The health systems studies	2
	The evidence based biomedical sciences	2
	Health policy	2
	Addiction studies	2
	Health economy	2
	Health philosophy and health ethics	2
	Statistical software applications in health researches	1
	Geriatric epidemiology	2
	Mental health	2
	Qualitative studies	2
	Environmental factors epidemiology	2
	Disaster epidemiology	2
	Total	15

*The MD/MPH students should select 15 units out of 23 offered units.

The MPH courses are compatible with medical courses. The number of units per MPH semester is 4-6. The courses are held in the afternoons and weekends.

Students in this program visit the research centers and health care providing centers based on the courses taken (e.g. visiting Drop-In Centers for students selecting addiction and health studies course or visiting HIV/AIDS non-governmental organizations for students passing the related courses). Furthermore, holding scientific meetings by experts in health sciences is another type of activity in MD/MPH program.

**Certification**

MPH degree is granted to the graduates of the MD/MPH program in addition to MD certificate. Graduates of this program can participate in the postgraduate studies in different Ph.D and medical specialties. MPH degree will be awarded to those who have successfully passed their medical courses. 

## Discussion

Health systems in the world are faced with various and changing issues. For example, communicable diseases were very important in the past; however, chronic and non-communicable diseases through epidemiological transition have become more serious in the recent decades. Presently, the significant influence of environmental factors, air pollution, occupational risk factors and social determinants of health on communities’ health has been identified. The MD/MPH program provides the graduates with enough knowledge to face with diverse and changing health-related issues and enable them to deal with these situations in the future (18, 25-26).

Based on this, the MD/MPH program is implemented in different universities in the world. Each of these programs has had different experiences and findings that indicate their effectiveness. For example, a study on the MD/MPH program at Tufts University showed that the implementation of MD/MPH program can better meet the changing needs of clinicians and their patients while also promoting the health of the public (10). Establishment of the MD/MPH program at the Texas' University led to students' high satisfaction as well as their vision in the medical field. The studies conducted on this program showed the influence of this program on the students' career and goals in the future (27). Another study conducted on the MD/MPH program at Columbia University indicated that students’ interest in passing health policy, international health and clinical prevention was the main reason of their interest in the MD/MPH program. The findings of the program showed that epidemiology, biostatistics, health policy, and health management were the most beneficial courses in the MD/MPH program. Based on the study, the intensive clinical training was the major reason of quitting MPH program in Columbia University. The students who studied MD/MPH in Columbia University did not differ greatly from their classmates in their specialty choices, but those who completed the MPH chose academic, governmental, and corporate practice settings more frequently than other young physicians, and devoted more time to non-clinical activities (28). In another research on the students’ attitudes towards MD/MPH program at Tulane University, the program led to the students’ broader perspectives on health issues. Catching more job opportunities was another benefit of the program (29).

In our study, MD/MPH program was designed through Delphi technique which is one of the most effective methods to reflect the individuals’ diverse ideas and opinions. In fact, designing/implementing committee of the program tried to localize the MD/MPH program model applied in other universities and increase the potential benefits of the program using Delphi method. For instance, the beginning time and the period of the program were localized according to the current medical education condition in SUMS. To illustrate, MD/MPH is a full-time program in some of the world universities and medical students have to quit their medical education for a period of time to pass MPH. However, this study showed that passing MPH program and medical education at the same time is more effective and practical in SUMS, a notion which was frequently mentioned by the Delphi team members. Moreover, entering the MPH program at the beginning of the physiopathology stage is the most suitable entry time for medical students regarding their knowledge gained through the passed medical courses at that point of time and also their medical studies in the future.

 This study had some limitations. Firstly, Delphi team members consisted of only experts of the field and not students. To confront this restriction, some research was conducted on MD/MPH students immediately after initiation of the program and some revisions were held based on the results. For example, the former voluntary courses in the program were then added by defining scientific writing (2 units) and academic writing (2 units) courses after doing some need assessment studies on MD/MPH students. Moreover, the Delphi team members were so busy and gathering research data through Delphi rounds was a time consuming process.

Consequently, MD/MPH program is implemented in SUMS presently and more than 100 medical students through three application periods have entered the program and are passing their courses continually. 

## Conclusion

In general, designing MD/MPH program in SUMS based on the existent models, compatible with educational program of this university and needs of national health system in Iran can be a beneficial measure towards promoting the students’ knowledge and theoretical/practical skills in both individual and social level.

At last, performing further research to assess the MD/MPH program and some cohort studies to evaluate the effect of this program on the students’ future professional life is recommended. 
